# Barriers and facilitators for treatment-seeking for mental health conditions and substance misuse: multi-perspective focus group study within the military

**DOI:** 10.1192/bjo.2020.136

**Published:** 2020-11-25

**Authors:** Rebecca Bogaers, Elbert Geuze, Jaap van Weeghel, Fenna Leijten, Dike van de Mheen, Piia Varis, Andrea Rozema, Evelien Brouwers

**Affiliations:** Tranzo, Scientific Center for Care and Wellfare, Tilburg School of Social and Behavioral Sciences, Tilburg University; and Brain Research and Innovation Centre, Ministry of Defence, the Netherlands; Brain Research and Innovation Centre, Ministry of Defence; and Brain Center Rudolf Magnus, Department of Psychiatry, University Medical Center Utrecht, the Netherlands; Tranzo, Scientific Center for Care and Wellfare, Tilburg School of Social and Behavioral Sciences, Tilburg University, the Netherlands; Strategic Military Healthcare Department, Ministry of Defence, the Netherlands; Tranzo, Scientific Center for Care and Wellfare, Tilburg School of Social and Behavioral Sciences, Tilburg University, the Netherlands; Department of Culture Studies, Tilburg School of Humanities and Digital Sciences, Tilburg University, the Netherlands; Tranzo, Scientific Center for Care and Wellfare, Tilburg School of Social and Behavioral Sciences, Tilburg University, the Netherlands; Strategic Military Healthcare Department, Ministry of Defence, the Netherlands

**Keywords:** Mental health conditions, substance misuse, treatment gap, stigma, military

## Abstract

**Background:**

Globally, millions are exposed to stressors at work that increase their vulnerability to develop mental health conditions and substance misuse (such as soldiers, policemen, doctors). However, these types of professionals especially are expected to be strong and healthy, and this contrast may worsen their treatment gap. Although the treatment gap in the military has been studied before, perspectives of different stakeholders involved have largely been ignored, even though they play an important role.

**Aims:**

To study the barriers and facilitators for treatment-seeking in the military, from three different perspectives.

**Method:**

In total, 46 people participated, divided into eight homogeneous focus groups, including three perspectives: soldiers with mental health conditions and substance misuse (*n* = 20), soldiers without mental health conditions and substance misuse (*n* = 10) and mental health professionals (*n* = 16). Sessions were audio-taped and transcribed verbatim. Content analysis was done by applying a general inductive approach using ATLAS.ti-8.4.4 software.

**Results:**

Five barriers for treatment-seeking were identified: fear of negative career consequences, fear of social rejection, confidentiality concerns, the ‘strong worker’ workplace culture and practical barriers. Three facilitators were identified: social support, accessibility and knowledge, and healthcare within the military. The views of the different stakeholder groups were highly congruent.

**Conclusions:**

Barriers for treatment-seeking were mostly stigma related (fear of career consequences, fear of social rejection and the ‘strong worker’ workplace culture) and this was widely recognised by all groups. Social support from family, peers, supervisors and professionals were identified as important facilitators. A decrease in the treatment gap for mental health conditions and substance misuse is needed and these findings provide direction for future research and destigmatising interventions.

## Background

Globally, millions are exposed to stressors at work that increase their vulnerability to develop mental health conditions and substance misuse (such as soldiers, policemen and doctors)^1^. Specifically, soldiers have an increased risk for mental health conditions and substance misuse after deployment^2^ and research shows that 60% of soldiers with mental health conditions and substance misuse, who could benefit from treatment, do not seek treatment,^3^ leading to a treatment gap for these issues. Leaving mental health conditions and substance misuse untreated poses a threat to sustainable employment through a higher risk for sick leave and unemployment.^4,5^ Beside negative consequences that affect well-being at an individual level, there are high economic costs involved when leaving these conditions untreated.^6^

In order to reduce the treatment gap, it is essential to examine causes of non-treatment-seeking. Multiple reviews have examined barriers and facilitators for treatment-seeking,^7–9^ with one of the main barriers being concern about stigma.^3,10^ There are different types of stigma, and in line with previous research^11^ the current study will focus on three types:

- public stigma – members of the general population endorse prejudice and discrimination against individuals with mental health conditions and substance misuse,^12^
- self-stigma – occurs when individuals with these conditions internalise the negative stereotypes and prejudices held by the general public,^13^ and
- structural discrimination – rules/regulations that either intentionally or unintentionally disadvantage individuals with mental health conditions and substance misuse.^14^

For example, US marines indicated being afraid that receiving treatment would cause them to be seen as weak (public stigma) and cause them to be treated differently in an unfair way (structural discrimination).^15^ One main facilitator has been found to be supportive leadership.^11^ Although research, both quantitative and qualitative, has been conducted on barriers and facilitators of treatment,^9^ it remains a complex phenomenon and more research is needed.^3,16^ Existing research has focused on the perspectives of soldiers with mental health conditions and substance misuse, largely ignoring perspectives of soldiers without these conditions and military mental health professionals,^7–9^ although they play a significant role in the decision to seek treatment. First, soldiers without mental health conditions and substance misuse problems potentially hold negative views (public stigma) and influence others in deciding to seek treatment.^17^ Second, they may develop these conditions themselves in the future, making it relevant to explore what would determine their treatment-seeking. Third, existing research has shown that generally, (civilian) mental health professionals hold stigmatising attitudes towards patients with substance misuse issues (for example perceive them as dangerous or responsible for their own substance misuse), which negatively influences treatment outcomes.^18^ Finally, military mental health professionals have influence on how mental healthcare is provided, and can potentially take away certain barriers. As they can be part of the solution, it is valuable to examine their perspectives.

## Aims

Using a multi-perspective approach can validate and extend earlier findings, providing more insight into the complex decision to seek treatment. The aim of the current study was to examine barriers and facilitators of treatment-seeking for mental health conditions and substance misuse in the military, using a qualitative approach from multiple perspectives. Specifically, the research questions were:

- What are the barriers and facilitators for treatment-seeking within the military, and
- What are the differences and similarities in the views between the three groups?

## Method

The COREQ-checklist, a guideline for reporting qualitative research, was used in reporting this study (see supplementary material 1, available at https://doi.org/10.1192/bjo.2020.136).^19^

### Setting

The study took place within the Dutch military – a military force with approximately 40 000 soldiers and no compulsory military service. Mental healthcare is organised internally and is available relatively close to a soldier's home. Soldiers can seek treatment for both mental health conditions and substance misuse and costs are covered by a military specific insurance. For ‘soft drugs’ (such as marijuana, hashish, sleeping pills) and alcohol, treatment is provided within the military, for ‘hard drugs’ (such as heroin, cocaine, amphetamine) soldiers are referred externally. A soldier can also individually decide to seek treatment outside of the military; however, these costs will not be covered by their insurance. As for the policies for substance misuse, there is a zero-tolerance policy for use of hard drugs, with the sanction being discharge. The use of alcohol is only prohibited during training and deployment. Use of soft drugs results in an official warning. However, when substance (mis)use is reported to a mental health professional, there are confidentiality agreements, and treatment is possible. When a soldier seeks treatment, their treatment and diagnosis are not reported to their supervisor. The soldier can decide whether he/she tells the supervisor about the mental health condition or substance misuse.

### Ethical considerations

Anonymity in reporting of results was guaranteed to all participants. All procedures were approved by the Tilburg School of Social and Behavioral Sciences Ethics Review Boards (approval number EC-2018.107) and the Military Ethics Review Board. The authors assert that all procedures contributing to this work complied with ethical standards of relevant national and institutional committees on human experimentation and with the Helsinki Declaration of 1975, as revised in 2008.

### Design

Qualitative research was chosen, as this is the preferable method for exploratory research when subject matters are complex or sensitive, which is the case for the current study.^20^ Specifically, focus groups were used as interaction among participants creates an in-depth understanding of complex subjects.^21^ Content analysis was done by applying a general inductive approach using ATLAS.ti-8.4.4 software.

### Participants

In total 46 people participated, divided over eight homogeneous focus groups. As the perspective of soldiers with mental health conditions and/or substance misuse was expected to be more elaborate, as they have lived experience, four groups were recruited for this perspective, and two for each of the other perspectives. Demographics can be found in Table 1. Two people signed up for the study, but dropped out because of illness.

**Table 1:** Participant characteristics in focus groups

Demographics	Soldiers with mental health conditions and/or substance misuse (groups 4, *n* = 20)	Soldiers without mental health conditions and substance misuse (groups 2, *n* = 10)	Military mental health professionals (groups 2, *n* = 16)
Age, years: mean (s.d.)	46.25 (8.32)	31.8 (10.30)	41.81 (10.29)
Male, *n* (%)	18 (90)	8 (80)	11 (69)
Married/living together, *n* (%)	19 (95)	6 (60)	11 (69)
Permanent contract, *n* (%)	20 (100)	8 (80)	16 (100)
Ranks, *n* (%)			
Staff officer	6 (30)	4 (40)	15 (94)
Non-commissioned officer	10 (50)	1 (10)	0 (0)
Corporals	0 (0)	1 (10)	0 (0)
Private	0 (0)	4 (40)	0 (0)
Civilian	0 (0)	0 (0)	1 (6)
Unknown	4 (20)	0 (0)	0 (0)
Branches of military, *n* (%)			
Army	11 (55)	2 (20)	N/A
Navy	4 (20)	5 (50)	N/A
Air force	2 (10)	1 (10)	N/A
Military police	3 (15)	0 (0)	N/A
Policy and support	0 (0)	2 (20)	N/A
Mental health condition,^a^ *n* (%)			
Post-traumatic stress disorder	6 (30)	N/A	N/A
Depression	5 (25)	N/A	N/A
Burnout	4 (20)	N/A	N/A
Attention-deficit hyperactivity disorder	2 (10)	N/A	N/A
Substance misuse	2 (10)	N/A	N/A
Personality disorder	2 (10)	N/A	N/A
Autism	1 (5)	N/A	N/A
Profession, *n* (%)			
Psychologist	N/A	N/A	7 (44)
Social worker	N/A	N/A	3 (19)
Mental health nurse	N/A	N/A	1 (6)
Chaplain	N/A	N/A	2 (13)
Occupational physician	N/A	N/A	1 (6)
Systemic family therapist	N/A	N/A	1 (6)
General practitioner	N/A	N/A	1 (6)

N/A, not applicable.

a. Total more than 100%, caused by two participants with a dual diagnosis.

### Procedure

Participants were approached through (a) flyers in waiting rooms of the mental health departments, (b) flyers at several military bases, (c) military psychologists who invited their patients, (d) adverts in a military newsletter, (e) personal contacts of one of the researchers, and (e) word-of-mouth between participants. After potential participants showed interest in participating (through email or telephone), they received an information letter and registration information. Participants were assigned to the specific focus groups based on whether they indicated having (had) mental health conditions and substance misuse or not (in the quotes below defined as the ‘with group’ and the ‘without group’, respectively) or being a military mental health professional. Written informed consent was obtained from all participants prior to participation in the focus groups.

First, participants filled out a demographic's questionnaire (including mental health diagnosis), and then the focus group leaders introduced themselves (names and research background). Focus groups were held in multiple rounds to facilitate iteration.^19^ All focus groups took place at military locations, lasted 2 h and were audio-recorded and transcribed verbatim. Additionally, the second focus group leader took notes. After every focus group, notes were reviewed, and if needed slight adjustments were made to the topic list to ensure sufficient attention was paid to all topics. No major new topics came up in the last focus group, indicating saturation.

All focus groups were facilitated by two (female) researchers (first author, R.B., MSc) and a coauthor (E.B. or A.R., both PhD), all with a background in psychology and health sciences and experienced in qualitative research. None of the researchers were actively involved in treatment of patients. The first author was familiar with two participants (both soldiers without mental health conditions and substance misuse) through a friend, but had no personal relationship with them. It was made clear that the mutual friend would not find out about their participation.

### Measurement

A topic list was developed based on existing literature. As this study was explorative, the aim was to see what barriers and facilitators for treatment-seeking the participants identified themselves, using open questions. However, when needed, probes based on current literature were used (for example ‘What role does a supervisor play in the decision to seek treatment?’).^7–9^ The same topic list was used for all focus groups, with slight adjustments that made questions applicable to participants in a specific group. The topic list was piloted among experts within the military. This topic list can be found in the supplementary material 2, available at https://doi.org/10.1192/bjo.2020.136.

### Analysis

Content analysis was done by applying a general inductive approach using ATLAS.ti (8.4.4) software.^22^ To ensure reliability, all transcripts were coded independently by the main researcher (R.B.) and a second member of the research team (E.B., E.G., J.v.W., A.R. or F.L.). Differences were discussed until consensus was reached, where about one-fifth of all codes were modified. Coders used an open, bottom-up, inductive coding style. Next, overarching categories were identified by one researcher, and checked by a second. In order to increase validity, multiple members of the research team identified the final categories. Analysis remained at a category level, in order to not lose valuable information by summarising at a theme level.

## Results

Five main categories of barriers were found:

- fear of negative career consequences,
- fear of social rejection,
- confidentiality concerns,
- the ‘strong worker’ workplace culture, and
- practical barriers.

There were three main categories of facilitators:

- social support,
- accessibility and knowledge,
- and healthcare within the military.

First the overarching results will be discussed, which will be followed by an examination of differences between the groups. For a full overview of the results, see the Appendix.

### Barriers

#### Fear of negative career consequences

All groups mentioned the fear for negative career consequences as a barrier to treatment-seeking. Participants indicated that seeking treatment could lead to (a) losing their job, (b) receiving negative differential treatment by not being allowed to do what they like most about their job, for example going on deployment, and (c) not being able to advance in their careers. The fear of losing their job applied to all mental health conditions and substance misuse, however, it appeared especially critical for substance misuse and addiction.

‘For us [air force] it is very clear that you will not be allowed to fly [if you have a mental health condition and substance misuse]. And that is what people love most about their job. So, they postpone seeking treatment.’ (male soldier, without group)

‘When I was in treatment [for post-traumatic stress disorder], I still kept my addiction a secret. I thought – if I tell them now – I will lose my job.’ (male soldier, with group):

#### Fear of social rejection

All groups also indicated fear of social rejection as a barrier, especially when mental health conditions and substance misuse were non-work related. First, there was fear of being rejected, literally being removed from the group, and of being seen as weak by peers.

‘You want someone who is capable of doing their job, and not some crazy person. On our deployment we removed someone from our group. Because he, well he was just a weird guy.’ (male soldier, without group)

Second, there was fear of social rejection by a supervisor. Participants indicated that a supervisor's negative attitude concerning treatment-seeking and mental health conditions and substance misuse in general, was an influence on a soldier's treatment-seeking.

‘During military training you notice that it is a no-go. The officers laugh about any form of mental health treatment. [….] There is a trend, it [mental health conditions and substance misuse] doesn't exists or isn't cool, and you will be rejected and removed from the group [if you have those conditions].’ (male, mental health professional)

#### Confidentiality concerns

All groups discussed confidentiality concerns associated with mental healthcare facilities forming a barrier for treatment-seeking. The fear of a breach of confidentiality was related to previous barriers, as this might lead to negative career consequences and/or social rejection. Because healthcare is organised within the military and is located at a military location, soldiers indicated that anonymity is lacking for soldiers who seek healthcare, and that soldiers are afraid to get recognised in waiting rooms.

‘I ran into one of my subordinates in the waiting room for the psychologist – that was uncomfortable.’ (male soldier, with group)

Participants also indicated a lack of a trusting relationship with the (mental) healthcare providers. Soldiers tended not to trust whether what they said to their (mental) healthcare provider was really confidential. Participants mentioned there was a high turnover of healthcare professionals within the military that stands in the way of having the time to build a trusting relationship with them.

#### Workplace culture

All groups spoke about the influence of the military workplace culture on the decision to seek treatment. They described this as a place where soldiers are expected to be strong, and confront challenges of any kind, rather than showing weakness or uncertainty, i.e. having a ‘can-do’ mentality. As a result, soldiers indicated seeing themselves as weak/a failure for having mental health conditions and substance misuse. This ‘can-do’ mentality also causes a failure to recognise the need for treatment. Additionally, they discussed the importance of self-management within the military workplace culture, and how this formed a barrier for treatment-seeking.

‘Soldiers are used to having control, and to then discover that they can't solve something themselves, […], well then shame comes into play. It is a form of failure.’ (male, mental health professional)

‘It started 4 years ago; I was getting some mental health complaints as a result of my first deployment. I was easily annoyed and had nightmares, which got worse. But you deny those symptoms to yourself. You just keep going and going.’ (male soldier, with group)

‘Most guys don't see it [alcohol misuse] as a problem, […] and before you can talk to them about it, well they should first realize [they have a problem]. This realization often never comes.’ (female soldier, without group)

Focus group leader: ‘What would you do if you would develop a depression?’

Soldier: ‘I would try to solve it by myself.’ (male, without group)

‘The focus is to always keep on going, take your own responsibility, you are trained in that way.’ (male, mental health professional)

As a result of wanting to solve things themselves, it also happens that help is provided within their own group. Soldiers want to protect each other from having to seek help outside of the group and its stigmatising consequences.

‘There are soldiers who are ill, but it won't show up in the system. Those soldiers also don't go into treatment, because they are kept in the shadows for a while. We take good care of each other.’ (male solider, with group)

[Giving example of how supervisors think] ‘There is also an aspect of ethics involved. Sh*t, I know someone used cocaine. Sh*t, I have to confront him. This will result in him losing his job. He has a wife and kids. There will be consequences. I don't want to see it. I don't see it.’ (male solider, with group)

#### Practical barriers

All groups indicated practical barriers for treatment-seeking. First, there is knowledge that help is available, but soldiers do not know who or where to go to for this help.

Focus group leader: ‘Would you know where to find help?’

Soldier: ‘Actually, no I would not.’ (male, without group)

Additionally, they mentioned that there is often a lack of time for treatment because of being understaffed and having a busy schedule.

[We would tell colleagues with mental health conditions and substance misuse] ‘You should not overreact, you just have to come with us [on training], because we need you. Shake it off and keep going.’ (male solider, without group)

### Facilitators

#### Social support to encourage treatment-seeking

Social support to encourage treatment-seeking was mentioned by all groups as a facilitator. Four sources of social support were mentioned to be important: family, peers, supervisors and mental health professionals.

‘My family was the reason for me [to seek help]. I was afraid to be judged at work, or miss a career opportunity. For me support did not come from work, but from my family.’ (male solider, with group)

‘Colleagues advise each other to go to a mental health professional. Patients tell others “you should really go to a mental health professional, I have also been, and it really helped me”.’ (female, mental health professional)

‘When a higher-ranking [soldier] shows subordinates that the healthcare system is important, this creates a different atmosphere for seeking treatment.’ (female, mental health professional)

‘It is really important to build a trusting relationship with a mental health professional first’. (male solider, with group)

#### Accessibility and knowledge

All groups discussed the importance of easily accessible healthcare, relatively short waiting lists and quick referrals. Additionally, they mentioned the importance of having knowledge of how the military mental health service works – that they know what help is available and who they should go to for help first.

#### Healthcare within the military

Two properties of the military healthcare system were mentioned as facilitators. First, participants mentioned the importance of mental health professionals who are familiar with and part of the military context – which is the case within the Dutch military. However, this was only the case for participants with mental health conditions, not for substance misuse. The latter indicated a preference for help for substance misuse outside the military out of fear for negative career consequences.

‘The mental health professional should be someone who understands you and has shared your experiences. Civilians, they have never been through it, you will just end up having to explain everything to them.’ (male solider, without group)

Second, doctor–patient confidentiality, which was mentioned as a barrier by some participants, but was also mentioned as an important facilitator. Soldiers discussed that it is wise to go to a mental health professional first and ask for advice, because they are obligated to keep your information confidential.

### Differences and similarities in views between groups

As can be seen from the Appendix, across the three different perspectives, the barriers and facilitators identified were very similar. All main categories of barriers and facilitators were identified by all the different groups. Besides the fact that the groups were very similar, some subcategories were not discussed in certain groups. For barriers, fear of social rejection by the supervisor was not mentioned by soldiers with mental health conditions and substance misuse; feelings of shame were not mentioned by soldiers without these conditions; and the failure to recognise need for treatment was not mentioned by mental health professionals. For facilitators, support from family was only mentioned by soldiers with mental health conditions and substance misuse; support from the supervisor was only mentioned by mental health professionals; and the preference for mental health professionals who are familiar with the military context, was not mentioned by the mental health professionals themselves.

## Discussion

### Main findings and comparison with findings from other studies

Although barriers and facilitators for help-seeking were discussed in stakeholder groups with three different perspectives, the findings yielded striking resemblances rather than differences. The main barriers and facilitators were recognised from all three perspectives (soldiers with and without mental health conditions and substance misuse, and mental health professionals), with only minor differences. The results showed that treatment is a complex decision, as has also been found in previous research.^16^ There is fear for negative career consequences and social rejection, and concerns surrounding confidentiality, the ‘strong worker’ workplace culture and practical barriers. On the other hand, social support, accessible care and having internal military mental healthcare all help soldiers in seeking treatment.

#### Stigma and treatment-seeking

The majority of barriers to treatment-seeking were related to different types of stigma. The fear for negative career consequences is related to structural discrimination, the fear of social rejection is related to public stigma, and the ‘strong worker’ workplace culture is related to self-stigma. Previous research showed mixed findings for the relationship between stigma and treatment-seeking, where some find a negative relationship,^11,23^ some find no relationship^3,17^ and others even find a positive relationship.^24^

Concerning the fear of negative career consequences, existing research, both quantitative and qualitative, has mainly focused on fear of differential treatment and lack of career advancement opportunities.^3,11,25^ However, fear of losing a job has received far less attention,^26^ whereas it is recognised by all stakeholders in the current study, especially for substance misuse. Additionally, interventions that have been implemented in other countries have largely ignored this concern.^26,27^ Fear of social rejection, the workplace culture, confidentiality concerns and practical barriers have all received attention in existing literature, both quantitative and qualitative.^3,10,26,28,29^

#### Social support issues

In the current study social support, both from family and the work environment, was found to be facilitating for treatment-seeking, which has also been found by other researchers.^29,30^ The importance of social support from the work environment (peers and supervisors) is directly related to the fear of social rejection being a barrier for treatment-seeking. The importance of social support in the decision to seek treatment is not surprising, as the military is known for high social cohesion.^30^ The other two facilitators, accessible care (including knowledge of where to find help) and preference for healthcare within the military, have been recognised by existing literature.^11,31^

#### Comparison of findings in the different groups

For the comparison of views, there was high agreement across the three stakeholder groups for all main barriers and facilitators for treatment-seeking. Existing research on this topic with similar findings only examined views of soldiers with mental health conditions and substance misuse.^3,7,9^ The high agreement across different perspectives implies that help-seeking actually does pose a risk for negative career consequences and social rejection, and that a key to closing the treatment gap can be found in taking measures to overcome these adverse outcomes from occurring.

As for differences between groups, two are noteworthy. First, supervisors’ attitude towards treatment and mental health conditions and substance misuse, both as barrier and facilitator, was not mentioned by soldiers with mental health conditions and substance misuse themselves. A possible explanation is that many soldiers with these issues mentioned that they had sought treatment because they had no other choice – their symptoms were too severe. Previous qualitative research also showed that soldiers seek treatment at a ‘crisis point’.^16,32^ It could thus be that a supervisor plays an important role when someone is contemplating seeking treatment, however, when symptoms become too severe, the supervisor's role becomes negligible. Nonetheless, existing research has shown that military leaders are influential for the decision to seek treatment,^11^ providing motive for further research into when and how military leaders influence the decision to seek treatment. Second, it is noteworthy that mental health professionals did not mention failure to recognise need for treatment as a barrier. This has been recognised as a barrier in existing studies, but has to our knowledge never been found within a population of military mental health professionals.^33^ This seems logical, as professionals will only see those who have recognised the need for treatment. However, better awareness of this can give guidance to mental health professionals in how they provide care and how they approach new patients.

### Strengths and limitations

The first strength of the present study was that multiple perspectives and types of mental health conditions and substance misuse that were included, creating a comprehensive and realistic view on the topic.^29,34,35^ Additionally, the sample included participants with an addiction, even though this is a sensitive topic within the military. Results showed that the fear of losing one's job was especially present for substance misuse, making the inclusion of participants with addiction valuable.

Second, this was, to the knowledge of the authors, the first study to examine barriers and facilitators for treatment-seeking within the Dutch military. As most studies have been done in the UK, Canada and the USA,^11^ the current study is a valuable addition to existing studies.

Third, measures were taken to ensure that participants would be able to speak freely about a relatively sensitive topic, which allowed for open and fruitful discussion. A safe and open environment was created in the current study by (a) using a diverse sample of soldiers from different armed forces to ensure participants were not familiar with each other, (b) asking participants to come in civilian clothes so as to not emphasise different ranks, and (c) ensuring focus group leaders could not influence participants’ careers as they were not part of the military.

The first limitation was the relatively low diversity in terms of age and rank between participants, with a majority of older and higher-ranking soldiers. It is possible that improvement has already been made to decrease the treatment gap within the younger generation of soldiers, but that this is not yet visible for the older generation. Findings of a British study also indicate that help-seeking is already improving.^36^

Second, risk of self-selection bias cannot be ruled out, as participants freely signed up for the study knowing what the study was about. Third, a limitation of using a focus group study is the lack of anonymity while discussing a sensitive topic. It is possible that participants only provided socially desirable answers. As discussed in the procedure section, the first author was familiar with two participants (both soldiers without mental health conditions and substance misuse), also forming a risk for socially desirable answers. However, as described above, several measures were taken to ensure confidentiality and allow participants to speak freely.

### Implications for practice and future research

The findings suggest that the treatment gap can be diminished by involving social support and encouragement from family, peers, supervisors and mental health professionals. Future research should investigate if interventions built upon increased support, and improved clarity about where and how to find help are effective in lessening the treatment gap. A starting point for clinical care would be to implement interventions that have already been proven effective in other countries that target social support, such as the peer-to peer programmes and programmes for military families implemented in the USA.^37,38^

Importantly, the study also highlighted the substantial barrier to treatment-seeking formed by stigma. Especially, the fear of negative career consequences (structural discrimination), social rejection (public stigma) and the ‘strong worker’ workplace culture (self-stigma) are important obstacles for seeking help. Clinical care should try to implement interventions targeting stigma that have proven to be effective in other armed forces, for example an online help-seeking stigma-reduction intervention that targets self-stigma.^39^

The high-level of agreement from different perspectives on these barriers implies that help-seeking actually does pose a risk for negative career consequences and social rejection, and that a key to closing the treatment gap can also be found in taking measures to overcome these adverse outcomes from occurring. Future intervention studies and policy measures should thus also focus on the barriers found in the present study, in order to decrease the treatment gap and improve health and sustainable employability.

## Data Availability

The data that support the findings of this study are available on request from the corresponding author, R.B. The data are not publicly available due to containing information that could compromise the privacy of research participants.

## Supplementary material

For supplementary material accompanying this paper visit http://dx.doi.org/10.1192/bjo.2020.136. click here to view supplementary material

## Appendix

### Barriers and facilitators to treatment-seeking for mental health conditions and substance misuse

**Table:**

Category	Subcategory	Soldiers with mental health conditions and substance misuse	Soldiers without mental health conditions and substance misuse	Mental health professional
Barrier 1: fear of negative career consequences	Losing employment (and subsequent financial concerns)	✓	✓	✓
	Negative differential treatment (different duties, no deployment)	✓	✓	✓
	Lack of career advancement	✓	✓	✓
Barrier 2: fear of social rejection	Fear of being rejected by/removed from the group	✓	✓	✓
	Fear of being seen as weak when having mental health conditions or substance misuse	✓	✓	✓
	Negative attitude of supervisor concerning treatment	×	✓	✓
Barrier 3: confidentiality concerns	Lack of anonymity when seeking help	✓	✓	✓
	Lack of trusting relationship with mental health professional	✓	✓	✓
Barrier 4: the ‘strong worker’ workplace culture	‘We can do it’ mentality	✓	✓	✓
	Failure to recognise need for treatment	✓	✓	×
	Preference for self-management	✓	✓	✓
	Feeling shame for having mental health conditions and substance misuse	✓	×	✓
	Help is provided within group in order to protect each other from stigma consequences	✓	✓	✓
Barrier 5: practical barriers	Lack of knowledge about who/where to go to for help	✓	✓	✓
	Lack of time for treatment (work obligations)	✓	✓	✓
Facilitator 1: social support to encourage treatment-seeking	Family/spouse support	✓	×	×
	Peer support (being sent to care by them)	✓	✓	✓
	Supervisor support (positive attitude supervisor)	×	×	✓
	Trusting relationship mental health professional	✓	✓	✓
Facilitator 2: accessibility and knowledge of where to get the right healthcare	Accessible healthcare (low key)	✓	✓	✓
	Relatively short waiting list, quick referrals	✓	✓	✓
	Knowledge of the healthcare system, and where to find help	✓	✓	✓
Facilitator 3: healthcare within the military	Mental health professionals that are familiar with and part of the military work context (except substance misuse)	✓	✓	×
	Anonymity in system through confidentiality by mental health professionals	✓	✓	✓

✓, Indicates that the subcategory was brought up and discussed by participants within a specific group of participants; ×, indicates that the subcategory was not mentioned within specific group of participants.
