# Barriers and facilitators for treatment-seeking for mental health conditions and substance misuse: multi-perspective focus group study within the military – CORRIGENDUM

**DOI:** 10.1192/bjo.2020.155

**Published:** 2021-02-08

**Authors:** Rebecca Bogaers, Elbert Geuze, Jaap van Weeghel, Fenna Leijten, Dike van de Mheen, Piia Varis, Andrea Rozema, Evelien Brouwers

**Keywords:** Mental health conditions, substance misuse, treatment gap, stigma, military, corrigendum

This article refers incorrectly to the affiliation of author Evelien Brouwers. The correct affiliation for this author is Tranzo, Scientific Center for Care and Wellfare, Tilburg School of Social and Behavioral Sciences, Tilburg University.
