# Supplementary material for: Barriers and facilitators for treatment-seeking for mental health conditions and substance misuse: multi-perspective focus group study within the military
Source: BJPsych Open. 2020 Nov 25;6(6):e146. doi: 10.1192/bjo.2020.136 (PMC7745246; doi:10.1192/bjo.2020.136)
Supplement: Supplementary file 1 [file S2056472420001362sup001.zip › bjo2000136_supplementarymaterial2_topiclist.docx]

| **Topic List** |
| --- |
| **Introduction to the topic.**  ‘How prevalent do you think mental health conditions and substance abuse are within the military?’  ‘What types of mental health conditions and substance abuse do you think are common within the military?’ |
| **Experiences/associations with the topic.**  ‘What are your experiences (associations) with the decision to seek treatment for MH/SAP?’ |
| **Barriers for treatment seeking.**  ‘What are barriers in the decision to seek treatment for mental health conditions and substance abuse?’  Example probes used when needed:  What are possible disadvantages of treatment seeking?  What would be practical barriers involved in treatment seeking?  How would treatment seeking influence someone’s career?  Are you aware of where to seek treatment for mental health conditions and substance abuse, and what care is available?  What is your view of the military mental health care service?  What role does a supervisor play in the decision to seek treatment? |
| **Facilitators for treatment seeking.**  ‘What are facilitators in the decision to seek treatment for mental health conditions and substance abuse?’  Example probes used when needed:  What are possible advantages of treatment seeking?  When and why would you seek treatment outside of the military?  Are you aware of where to seek treatment for mental health conditions and substance abuse and what care is available? What is your view of the military mental health care service?  What role does someone’s social environment play in the decision to seek treatment? |
| **Future needs as a closure of the topic.**  ‘What is needed in the future to assist soldiers with the decision to seek treatment for mental health conditions and substance abuse within the military?’. |
| ***Note:*** *Per focus group the questions were formulated slightly differently to make them suitable for the participants of that specific focus group. This means that for the military personnel with mental health conditions and substance abuse the questions were directed at their own experiences, for military personnel without mental health conditions and substance abuse the questions were directed at their experiences in general and with colleagues, and for professionals who provide mental health care the questions were directed at their experiences with the people they provide care for. All questions were asked in Dutch during the focus group.* |
